# Revisiting Hepatic Artery Infusion Chemotherapy in the Treatment of Advanced Hepatocellular Carcinoma

**DOI:** 10.3390/ijms222312880

**Published:** 2021-11-28

**Authors:** Ching-Tso Chen, Tsung-Hao Liu, Yu-Yun Shao, Kao-Lang Liu, Po-Chin Liang, Zhong-Zhe Lin

**Affiliations:** 1 Department of Oncology, National Taiwan University Hospital Hsinchu Branch, Hsinchu 300195, Taiwan; robarroyce@gmail.com; 2 Department of Oncology, National Taiwan University Hospital, Taipei 100225, Taiwan; tsunghao.liu@gmail.com (T.-H.L.); yuyunshao@gmail.com (Y.-Y.S.); 3 Graduate Institute of Oncology, College of Medicine, National Taiwan University, Taipei 100233, Taiwan; 4 Department of Medical Imaging, National Taiwan University Hospital, Taipei 100225, Taiwan; kaolangliu@gmail.com; 5 Department of Medical Imaging, National Taiwan University Cancer Center, Taipei 106328, Taiwan; 6 Department of Medical Imaging, National Taiwan University Hospital Hsinchu Branch, Hsinchu 300195, Taiwan; 7 Department of Internal Medicine, College of Medicine, National Taiwan University, Taipei 100233, Taiwan; 8 Department of Medical Oncology, National Taiwan University Cancer Center, Taipei 106328, Taiwan

**Keywords:** hepatocellular carcinoma, intra-arterial chemotherapy, targeted therapy, immunotherapy

## Abstract

Hepatic artery infusion chemotherapy (HAIC) is a well-established and common treatment for advanced hepatocellular carcinoma (HCC), particularly in East Asia. However, HAIC is not recognized internationally. Although several trials have demonstrated the safety and efficacy of HAIC, evidence corroborating its overall survival (OS) benefits compared with standard treatments is insufficient. Nevertheless, HAIC may provide prominent benefits in selected patients such as patients with portal vein thrombosis or high intrahepatic tumor burden. Moreover, HAIC has been combined with several therapeutic agents and modalities, including interferon-alpha, multikinase inhibitors, radiation therapy, and immunotherapy, to augment its treatment efficacy. Most of these combinations appeared to increase overall response rates compared with HAIC alone, but results regarding OS are inconclusive. Two prospective randomized controlled trials comparing HAIC plus sorafenib with sorafenib alone have reported conflicting results, necessitating further research. As immunotherapy-based combinations became the mainstream treatments for advanced HCC, HAIC plus immunotherapy-based treatments also showed encouraging preliminary results. The trials of HAIC were heterogeneous in terms of patient selection, chemotherapy regimens and doses, HAIC combination agent selections, and HAIC technical protocols. These heterogeneities may contribute to differences in treatment efficacy, thus increasing the difficulty of interpreting trial results. We propose that future trials of HAIC standardize these key factors to reveal the clinical value of HAIC-based treatments for HCC.

## 1. Introduction

**Figure 1:**
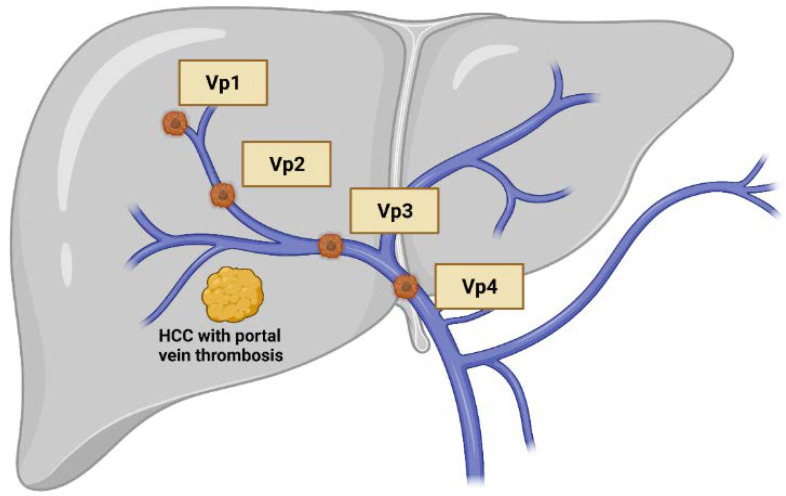
Classification of macrovascular invasion of hepatocellular carcinoma, including portal vein thrombosis and/or tumor invasion. Vp1: the third order branch or portal vein; Vp2: the second order branch of portal vein; Vp3: the first order branch of portal vein; Vp4: the main trunk of portal vein. Created with BioRender.com.

Hepatic artery infusion chemotherapy (HAIC) is a treatment modality for advanced hepatocellular carcinoma (HCC). HAIC entails infusing chemotherapeutic agents directly into hepatic tumors through the percutaneous catheterization of feeding arteries. Because HCC tumors are primarily supplied by the hepatic arteries, HAIC provides a higher intratumoral concentration of chemotherapeutic agents and avoids the first-pass effect, theoretically yielding greater treatment efficacy and less hepatocellular injury [1]. These chemotherapeutic agents subsequently went through the body by circulation and also offered systemic anti-tumor effect but with less concentration advantage. Therefore, HAIC is basically a systemic treatment with more prominent locoregional efficacy. These peculiar features make HAIC distinct from other transarterial therapeutic approaches for HCC, such as transarterial chemoembolization (TACE) and selective internal radiation therapy (SIRT), which yield locoregional efficacy only and failed to provide survival benefit for patients with advanced HCC [2,3,4]. Furthermore, TACE is considered as relative contraindicated in patients with portal vein thrombosis (PVT), since reduced blood supply in both portal vein system and hepatic arteries may cause substantial hepatocyte injury, especially for Vp3/4 thrombosis (Figure 1). In contrast, HAIC can be performed safely in these patients.

HAIC has been utilized for advanced HCC more commonly in East Asia than in other regions of the world. Because viral hepatitis is endemic in East Asia, the region is among those with the highest disease burden for HCC [5], exhibiting distinct features in terms of epidemiology, etiology, diagnostic modalities, and treatment patterns. Many Asian HCC treatment guidelines adopt a more aggressive strategy for the use of HAIC [6,7,8,9], which is not yet recognized by many international organizations such as the National Comprehensive Cancer Network (NCCN) [10] or the European Society for Medical Oncology (ESMO) [11]; the under-recognition of this therapy is due to a lack of proven survival benefits from well-designed, randomized controlled trials in comparison with current standard treatments. In this review, we revisit current evidence regarding HAIC treatment for advanced HCC and assess its potential role in HCC treatment.

## 2. HAIC Monotherapy

HAIC has long been reported as a potential therapy for advanced HCC [12]. Before the advent of sorafenib, advanced HCC was often most effectively treated with supportive care, antiangiogenesis agents such as thalidomide [13], or chemotherapy. These treatments conferred limited objective response rates (ORR), ranging from 0% to 21%, and were associated with a risk of high rates of hematological toxicity [13,14,15,16]. By contrast, HAIC conferred higher ORRs, ranging from 5% to 71% (Table 1), and lower systemic toxicity [1]. A nationwide registry study in Japan compared HAIC treatment with no active treatment for patients with advanced HCC; the study revealed that HAIC was associated with improved overall survival (OS) compared with the most effective supportive care (median survival, 14.0 vs. 5.0 months; hazard ratio [HR], 0.48; *p* < 0.001) [17]. Other retrospective studies have also reported higher efficacy of HAIC compared with transcatheter arterial chemoembolization (TACE) or systemic chemotherapy for advanced HCC [18,19].

As a result of the SHARP clinical trial [20] and associated Asia-Pacific trials [21], sorafenib became the first standard systemic treatment with improved OS for advanced HCC compared with placebos. Several small-scale studies have subsequently investigated whether HAIC can yield superior benefits over sorafenib for patients with advanced HCC. Such studies have generally reported that HAIC demonstrated higher ORRs than sorafenib did, but they could not draw definite conclusions regarding OS (Table 1) [22,23,24,25,26,27,28]. In the prospective SCOOP-2 Phase 2 trial comparing HAIC with sorafenib, HAIC was even associated with a numerically shorter OS compared with sorafenib (median survival, 10.0 vs. 15.7 months, *p* = 0.78). Additionally, HAIC antitumor effects on extrahepatic spread (EHS) were not specifically reported, but it was considered theoretically attenuated. Thus, HAIC monotherapy lacks sufficient evidence as a standard first-line therapy for advanced HCC.

Regarding second-line treatments and beyond, HAIC has not been directly compared with other second-line systemic therapeutic agents such as regorafenib, cabozantinib, and ramucirumab. HAIC after failure of sorafenib or other first-line treatments was reported to be effective and well tolerated, with a remarkable ORRs of approximately 30%, even in patients unsuitable for regorafenib treatment [29,30,31].

Selected patient populations may, however, gain greater benefit from HAIC. Many investigators have administered HAIC to patients with macrovascular invasion (MVI), a subgroup with inferior prognosis and required prompt treatment response. Retrospective studies focusing on patients with PVT have revealed that patients receiving HAIC had a longer OS compared with those receiving sorafenib treatment [22,28]. HAIC also provided survival benefits for large HCC as shown in retrospective studies [32,33], and also in a randomized Phase 3 study comparing HAIC and TACE in large (>7 cm) intermediate HCC [34]. Adverse events of HAIC in these studies were relatively low [32,34]. At the 2021 American Society of Clinical Oncology conference, Lyu et al. presented the results of FOHAIC trial comparing first-line HAIC with sorafenib in advanced HCC mainly with MVI and high tumor burden; they reported, for the first time in a prospective Phase 3 study, that HAIC could lead to a longer OS than sorafenib could (median survival, 13.9 vs. 8.2 months, *p* < 0.001) [35]. These study results support the efficacy of HAIC in patients with MVI or with large intrahepatic tumor burden.

Another area for HAIC monotherapy is in patients with poor liver function reserve, such as those with Child–Pugh (CP) Class B or C cirrhosis [6]. For such patients, systemic treatment choice is still very limited because most therapeutic modalities for advanced HCC were developed for patients with adequate liver function. The CP-B cohort in the CheckMate-040 trial [36] exhibited an attenuated ORR (10%) for nivolumab monotherapy, which was only half that observed for the CP-A cohort. Two retrospective studies have revealed survival benefits of HAIC over sorafenib treatment for CP-A and selected CP-B group [26,37], although such benefits were not consistently observed in other retrospective studies [28,38]. Terashima et al. [39] published a notable retrospective study of patients receiving sorafenib or HAIC and discovered that more patients receiving HAIC exhibited sustained or improved liver function after four weeks of treatment compared with patients receiving sorafenib (72% vs. 50%, *p* = 0.006). This result further indicates that HAIC may minimize injury to normal hepatocytes and possibly improves liver function by reducing tumor burden. Correspondingly, Liu et al. [40] reported a patient of advanced HCC with CP-C who received HAIC treatment. The patient had a good partial response and his liver function reserve also improved to CP-A gradually. Therefore, HAIC may be considered as a potential first-line treatment for patients withpoor liver function reserve.

## 3. HAIC-Based Combination Therapy

The following characteristics of HAIC render it a suitable candidate for combination with other antineoplastic agents for advanced HCC: it is associated with fewer systemic adverse events compared with intravenous chemotherapy, and its cytotoxic mechanism is distinct from those of other HCC therapeutic modalities. Several studies have explored potential HAIC-based combination strategies (Table 2).

### 3.1. HAIC Plus Subcutaneous Interferon-Alpha

Subcutaneous or intramuscular interferon-alpha (IFN-α) has been used in combination with intravenous chemotherapy for advanced HCC to enhance antitumor activity [55]. Subcutaneous IFN-α has also been combined with HAIC, resulting in higher ORRs than those achieved with HAIC alone, although the survival benefit of this combination is inconclusive [42,56,57]. However, a randomized Phase 2 trial comparing HAIC with or without IFN-α showed inferior OS for the group treated with the HAIC–IFN-α combination [43]. Because of such inconsistencies between study findings, IFN-α has not been routinely used in combination with HAIC.

### 3.2. HAIC Plus Multikinase Inhibitors

HAIC has been combined with sorafenib to leverage the synergistic effects of the combination. A randomized Phase 2 trial was conducted to compare HAIC plus sorafenib with sorafenib alone as a first-line therapy for patients with CP score of up to B7; the trial demonstrated that HAIC plus sorafenib resulted in a higher ORR (21.7% vs. 7.3%) and longer OS (median survival, 10.6 vs. 8.6 months, *p* = 0.031) [44]. Subsequently, Kudo et al. [45] conducted the SILIUS trial, a multicenter randomized Phase 3 trial comparing frontline use of sorafenib with or without HAIC, and confirmed a higher ORR and longer time to progression (TTP) in the combination group, but the OS were similar between two groups. They also conducted a subgroup analysis and revealed the combination therapy yielded longer OS than sorafenib treatment did in patients with Vp4 PVT. He et al. [47] reported another randomized Phase 3 trial comparing sorafenib with or without HAIC in 2019 in patients with PVT (Vp4: 37%); the results showed that patients treated with the combination therapy exhibited more favorable outcomes, including higher ORRs and longer OS periods (median survival, 13.4 vs. 7.1 months; HR 0.35; *p* < 0.01). Although these two studies have reported opposite results regarding the effects of first-line HAIC combination, they differed in several aspects. First, they enrolled different patients: all patients enrolled in the study by He et al. had PVT, whereas only 63.2% of those in the study by Kudo et al. had PVT. Hepatitis B virus–related HCC was less prevalent in the study by Kudo et al. (23.4%) than in the study by He et al. (80%). Second, He et al. administered an oxaliplatin-based regimen, modified FOLFOX6, every 3 weeks, which is also a common intravenous chemotherapy regimen for advanced HCC in China; by contrast, the regimen in the SILIUS trial was cisplatin plus 5-fluorouracil (5-FU) every 4 weeks. Because of inherent differences between oxaliplatin and cisplatin, the use of these two platinum-based chemotherapeutic modalities may result in different synergistic effects with sorafenib [58]. Third, He et al. used repeated intra-arterial catheterization, which allows for the adjustment of the microcatheter tip position and the re-embolization of newly developed gastroduodenal collateral arteries. These differences may contribute to the different OS results in these two trials. In summary, HAIC combined with sorafenib could provide favorable ORR and may provide OS benefits. Further research should be conducted to explore the optimal chemotherapeutic agents, protocol procedures, and target patient populations.

Data regarding the combination of HAIC with lenvatinib are limited. A retrospective study of 24 patients treated with HAIC plus standard-dose lenvatinib reported an encouraging ORR of 58% and a disease control rate of 79% [48]. Additional prospective studies of the combination of HAIC and lenvatinib are ongoing.

### 3.3. HAIC Plus Radiation Therapy

HAIC combined with radiation therapy (RT) has also been extensively investigated, particularly in subgroups of patients with PVT. Han et al. [51] conducted a small-scale single-arm pilot study of three-dimensional conformal RT followed by HAIC for HCC; they observed an ORR of 45% with manageable adverse events. Investigators from Hiroshima University, Japan, have published a series of retrospective studies comparing HAIC plus RT with HAIC alone, focusing on patients with PVT. Their results revealed impressive ORRs in the HAIC-RT combination arm, but no significant survival benefits were observed [52,53]. Furthermore, Kodama et al. [54] retrospectively reviewed the effects of HAIC plus RT compared with treatment with sorafenib in patients with major PVT (Vp3/4) by using case–control matching analysis. The HAIC-RT combination group demonstrated more favorable clinical outcomes, including OS (median survival, 9.9 vs. 5.3 months, *p* = 0.002) and progression-free survival (median survival, 3.9 vs. 2.1 months, *p* = 0.048). The findings of these studies indicate that HAIC plus RT may yield favorable ORRs and survival benefits; nevertheless, evidence from prospective randomized controlled studies is still unavailable.

### 3.4. HAIC Plus Immunotherapy

Immune checkpoint inhibitor–based combinations have changed the treatment paradigm for advanced HCC [59,60] and are likely to remain the cornerstone of systemic treatment in the next few years. The IMbrave150 trial compared treatment with atezolizumab plus bevacizumab and treatment with sorafenib; they reported an impressive ORR of 30% and an unprecedented OS benefit for the combination treatment over sorafenib (median survival, 19.2 vs. 13.4 months, HR 0.66) [60,61]. Several ongoing Phase 3 trials testing immune checkpoint inhibitors in combinations with other immuno-oncology agents or multikinase inhibitors (MKIs) are ongoing.

Chemotherapeutic modalities have been proved to be synergistic with anti-PD1/PD-L1 antibodies in several cancers, such as those of the lung and breast [62,63]. HAIC may also induce substantial local immune modulation in the intrahepatic tumor microenvironment of HCC. Whether HAIC plus PD1/PD-L1 blockade would have synergistic effects warrants further investigations. Preliminary results of early phase trials of PD-1 blockade plus MKIs have been promising [59], and investigations of triplet therapy, namely anti-PD-1, MKIs, and HAIC, are ongoing. Gu et al. [49] reported a single-center experience for six patients who received HAIC combined with apatinib and toripalimab as the first-line treatment for advanced HCC. All six patients responded to treatment (ORR, 100%), and three of the patients (50%) exhibited complete responses. He et al. [50] presented a retrospective study in which 71 patients underwent treatment involving a combination of HAIC, lenvatinib, and toripalimab; they reported a high ORR (59%) after treatment. These encouraging results support further research on HAIC combined with other immune-based therapeutic agents.

In summary, many studies have shown positive signs for HAIC combination treatments. In particular, for patients with major PVT, HAIC plus sorafenib provided a longer OS [45,47]. Regarding the combination of HAIC with other therapeutic modalities, HAIC plus RT or PD-1/PD-L1 blockade also demonstrated promising results [49,50,52,53,54]. We believe these HAIC-based combination treatments will become the dominant trend in clinical practice and clinical trials.

## 4. Potential Obstacles to Prospective HAIC Clinical Trials

Numerous retrospective studies on HAIC for advanced HCC are available, but prospective randomized trials are considerably fewer and are heterogeneous in terms of patient populations, chemotherapy regimens, and HAIC techniques. Such heterogeneity may lead to inconclusive results regarding specific outcomes such as OS.

### 4.1. Heterogeneous Patient Populations

Studies on HAIC have included populations with various degrees of intrahepatic tumor burden, including the possible presence of PVT/EHS. Studies on patients with PVT who received MKI treatment revealed that these patients had poor outcomes after treatment [20,21]. By contrast, HAIC was reported to be associated with encouraging ORRs (24 to 71%) and OS (7.1 to 30.4 months) in this group of patients (Table 1). Moreover, some studies have focused on patient subgroups with major PVT (Vp3/Vp4), and HAIC, applied alone or in combination with other treatment modalities, still demonstrated considerable efficacy and safety [22,38,53]. He et al. [47] recently revealed that HAIC plus sorafenib provided superior outcomes than did sorafenib in patients with HCC with PVT. By contrast, the SILIUS study which tested a similar combination strategy, enrolled a more heterogeneous group of patients and only 59.5% of whom had PVT. The inconsistency between the study results may partly be due to differences in patient populations. The importance of patient selection is further emphasized by the FOHAIC study, which reported that HAIC monotherapy yielded superior OS than did sorafenib in patients with MVI or large intrahepatic tumor burdens [35].

The presence of EHS would affect HAIC outcomes. Because HAIC has less therapeutic efficacy to extrahepatic tumors, it is suitable only for patients with limited or indolent EHS. Ueshima et al. [37] conducted a nationwide registry study in Japan by comparing HAIC with sorafenib in 2004 patients. Their subgroup analysis revealed that patients with MVI and without EHS who received HAIC had a significantly longer OS compared with those who received treatment of sorafenib. By contrast, patients with EHS and without MVI who received sorafenib treatment had longer OS than did those who received HAIC.

Previous TACE is another factor that may sabotage HAIC efficacy. This embolization would compromise original hepatic arterial supply to HCC tumors, and promote blood supply from the portal vein or extrahepatic collateral arteries [64], thus potentially attenuating response to HAIC treatment. Hatooka et al. [27] retrospectively compared HAIC and sorafenib treatment in a more specific population of patients with CP-A but without EHS who were refractory to TACE. Their results showed favorable OS in the sorafenib group. In summary, HAIC may confer the greatest benefit in patients who have PVT or a large hepatic tumor burden, who with no or limited EHS, and who are not refractory to TACE.

### 4.2. Diverse Chemotherapeutic Regimens

A standard chemotherapy regimen for HAIC has yet to be established, increasing the challenge of interpreting trial results. Cisplatin and 5-FU were the most commonly used chemotherapeutic agents with various infusion protocols. Other commonly used chemotherapeutic agents included oxaliplatin, carboplatin, epirubicin, and etoposide. The ORRs of these chemotherapies ranged from 5% to 71% in previous reports, with complete response rates of 1% to 5% [22,23,28,42,54]. Although no single regimen has been reported to demonstrate superiority over the others, differences may still exist among different regimens and various protocols. For example, doublet chemotherapy appeared to be associated with higher ORRs (30–40%) [26,44,46] compared with platinum alone (20–30%) [22,23,28,54]; moreover, regimens with higher doses (e.g., 5-FU at a total dose of >2000 mg/m2 or modified FOLFOX6) [23,42,47,48,49] probably engendered higher ORRs than did those with low doses of 5-FU (40–70% vs. 30–40%) [22,28,38,54]. Investigators in China have reported encouraging results with consistent use of modified FOLFOX6 as an HAIC regimen, particularly when used in combination with PD-1/PD-L1 blockade [47,48,49,50]. Notably, two Phase 3 trials, ATTRACTION-4 [65] and CheckMate-649 trials [66], which both compared oxaliplatin-based chemotherapy with or without PD-1 blockade in advanced gastric cancer, showed longer OS in the combination arm. The Keynote-062 trial [67], another Phase 3 trial, used cisplatin-based chemotherapy plus PD-1 blockade in the experimental arm and yielded similar OS compared with chemotherapy control arm. Whether oxaliplatin produces more synergistic effects with immunotherapy than cisplatin does remains unclear. Therefore, the potential distinct immune modulation effects of different chemotherapeutic agents warrant further investigation in HAIC.

### 4.3. Repeated Catheterization versus Implantable Port-Catheter Systems

Two different percutaneous arterial access approaches have been used for HAIC: the first approach entails the use of implantable port-catheter systems, and the second approach involves repeated hepatic artery catheterization. Implantable systems were commonly applied in previous decades [68]. Traditionally, these systems were surgically implanted under general anesthesia, but recently, they have also been implanted through minimally invasive procedures [69]. For implantable port-catheter systems, the HAIC approach is more convenient for both patients and physicians, but the use of implanted devices also increases the risk of infection and vascular complications. By contrast, repeated percutaneous catheterization offers the opportunity to reposition of the microcatheter tips in response to possible developments or changes in tumor angiogenesis. However, repeated invasive procedures are also accompanied by risks such as catheter occlusion, hepatic artery obstruction, hematoma, and puncture site infection [70]. Vascular complication rate was approximately 10% in implanted port system and was reported to be less in repeated invasive procedures [47]. We also observed a trend toward the use of repeated catheterization approach in recent HAIC trial designs [44,46,47,53,54].

### 4.4. Insufficient Technical Standardization

Techniques and protocols for HAIC have yet to be standardized. The efficacy and safety of HAIC depend substantially on the quality of vascular redistribution. The success rate of vascular redistribution is generally high (approximately 80%) [71]. Extrahepatic arterial flow into the liver, most commonly from the right inferior phrenic artery, may be an obstacle to redistribution [71,72]. Yamagumi et al. [72] reported that embolization of this extrahepatic artery may contribute to a successful redistribution. However, the protocols of redistribution are not standardized among different geographical regions and institutions.

Regarding port-catheter system implantation, different technical protocols vary in terms of the following details; arterial access site [70], catheter tip position [73], catheter tip fixation with glue or coil [74], and type of coils used for embolization [75]. Some investigators also incorporated lipiodol infusion into HAIC treatment [23,76,77]. In the design of future trials for HAIC, standardizing technical protocols among different centers is crucial to avoid biased outcomes.

### 4.5. Lack of Industry Incentive for Conducting Trials

The patents for the main chemotherapeutic agents used in HAIC, including cisplatin, oxaliplatin, 5-FU, and epirubicin, have expired, thus weakening the motivation for the pharmaceutical industry to sponsor HAIC studies. All the aforementioned prospective trials were not sponsored by pharmaceutical companies but by academic or governmental institutions. Such sponsorship may limit the scale of these trials and the incentives for further investigations. Several ongoing studies exploring the efficacy of HAIC in combination with novel anticancer agents, such as lenvatinib (Clinicaltrial.gov: NCT04135690), apatinib (NCT03775395), camrelizumab (NCT04479527), and toripalimab (NCT04191889), have also been initiated by the investigators themselves.

## 5. Conclusions

Many studies have demonstrated the potent antitumor efficacy of HAIC for advanced HCC. HAIC may also yield survival benefits over other systemic therapies such as sorafenib treatment, especially in patients with PVT or with high intrahepatic tumor burden. However, because of insufficient corroborating evidence from randomized Phase 3 trials, HAIC is underrecognized as a standard therapy for advanced HCC. Future research on HAIC must focus on patient selection, chemotherapy regimen choice, technical protocol standardization, and potential combinations with other therapeutic agents, to reveal the value of HAIC in current advanced HCC treatment. HAIC in combination with other therapeutic agents, especially immunotherapy-based regimens, had showed encouraging preliminary results and is likely to play a more important role in the future.

## Figures and Tables

**Table 1:** Selected studies on HAIC versus sorafenib as the first-line treatment for advanced HCC.

Group	Study Type/Characteristics	Patient Number	Regimen	CP-B (%)	HBV (%)	PVT (%)	EHS (%)	ORR (%)	OS (Months)	*p*-Value (OS)
Song et al. [28]	Retrospective PVT	50	Cisplatin 60 mg/m^2^, Day 2 5-FU 500 mg/m^2^, Days 1–3 +/− Epirubicin 35 mg/m^2^, Day 1 (every 3–4 weeks)	10.0	88.0	100	13.0	24.0	7.1	0.011
		60	Sorafenib	21.7	68.3	100	35.0	13.3	5.5	
Hatooka et al. [27]	Retrospective Refractory to TACE	65	Cisplatin 6 mg/m^2^, Days 1–5, 8–12 5-FU 300 mg/m^2^, Days 1–5, 8–12 * (every 4 weeks)	0	23.1	35.4 (Vp3–4)	0	12.0	8.0	0.021
		58	Sorafenib	0	22.4	10.3 (Vp3–4)	0	6.0	15.0	
Moriguchi et al. [22]	Retrospective Vp3–4	32	Cisplatin 10 mg/m^2^, Day 1; 5-FU 250 mg/m^2^, Days 1–5 (weekly for 4 weeks, then only Day 1 per week)	0	37.5	100	21.9	31.3	10.3	0.009
		14	Sorafenib	0	28.6	100	35.7	0	4.0	
Nakano et al. [23]	Retrospective With MVI, without EHS	44	Cisplatin 50 mg/m^2^ in 5–10 mL lipiodol, Day 1 5-FU 1500 mg/m^2^ for 5 day for 2 weeks then cisplatin 25–30 mg/m^2^ + 5FU 500–1000 mg/m^2^ (ever 2 weeks)	0	14.0	100	0	71.0	30.4	<0.001
		20	Sorafenib	0	25.0	100	0	10.0	13.2	
Kodama et al. [25]	Retrospective No EHS	150	Cisplatin 6 mg/m^2^, Days 1–5, 8–12 5-FU 300 mg/m^2^, Days 1–5, 8–12 (every 4 weeks)	0	25.3	73.3	0	32.0	10.0	0.007
		134	Sorafenib	0	16.4	29.1	0	4.0	19.0	
Lyu et al. [24]	Retrospective HAIC for patients who refused sorafenib	180	mFOLFOX 6 (HAIC) (every 3 weeks)	0	86.7	54.4	60	29.4	14.5	<0.001
		232	Sorafenib	0	80.2	55.6	58.6	3.0	7.0	
Kondo et al. [26]	Randomized Phase 2 (CP-A to B7)	35	Cisplatin 65 mg/m^2^, Day 1 (every 4–6 weeks)	11.4	8.6	60.0	28.6	14.3	10.0	0.780
		33	Sorafenib	12.1	12.1	66.7	24.2	9.1	15.2	
Ahn et al. [38]	Retrospective VP4	38	Cisplatin 60 mg/m^2^, Day 1 5-FU 500 mg/m^2^, Days 1–3	29.0	86.8	100	5.3	5.2	10	0.150
		35	Sorafenib	31.0	69.0	100	46	0	6.4	
Ueshima et al. [37]	Retrospective Cohort 1 with MVI, Without EHS	270	Cisplatin + 5FU or 5-FU or cisplatin (detail of regimens were not reported)	36.9	23.0	100	0	NR	10.6	0.475
		263	Sorafenib	16.0	21.3	100	0	NR	9.1	
Zaizen et al. [41]	Retrospective Propensity score-matched	83	Cisplatin 65 mg/m^2^, Day 1 (every 8–12 weeks)	36.1	7.2	14 (MVI)	0	NR	15.6	0.016
		83	Sorafenib	28.9	8.4	11(MVI)	0	NR	11.0	
Lyu et al. [35]	Randomized Phase 3	130	mFOLFOX 6 (HAIC) every 3 weeks	NR	NR	NR	NR	NR	13.9	<0.001
		132	Sorafenib	NR	NR	NR	MR	NR	8.2	

aHCC: advanced hepatocellular carcinoma; CP: Child–Pugh classification; EHS: extrahepatic spread; HAIC: hepatic arterial infusion chemotherapy; HBV: hepatitis B virus; IFN-α: interferon-alpha; MVI: macrovascular invasion; NR: not reported; ORR: overall response rate; OS: overall survival; PVT: portal vein thrombosis; TACE: transcatheter arterial chemoembolization; VP3: right/left portal vein; VP4: main portal vein; 5-FU: 5-fluorouracil. * 57% patients received 5-FU plus IFNα.

**Table 2:** Selected studies on HAIC combinations as first-line treatment for advanced HCC.

Group	Study Design	Patient Number (N)	Regimen	CP-B (%)	HBV (%)	PVT (%)	EHS (%)	ORR (%)	OS (Months)	*p*-Value (OS)
**INF-α**										
Sakon et al. [42]	Phase 2 single arm VP3–4, no EHS	11	5-FU 450–500 mg/m^2^, Days 1–5 INF-α5MU qW1,3,5	54.5	36.4	100	0	72.7	8.0	
Eun et al. [43]	Retrospective single arm	31	HAIC: 5-FU 750 mg/m^2^, cisplatin 25 mg/m^2^, Days 1–4 INF-α 3MU Days 1–4, then QOD	19.4	83.9	100	NR	19.4	4.0	0.353
		21	HAIC alone: 5-FU 750 mg/m^2^, cisplatin 25 mg/m^2^, Days 1–4	19.0	85.7	100	NR	42.9	7.0	
**Sorafenib**										
Ikeda et al. [44]	Randomized Phase 2 CPS-A, B7	65	Cisplatin 65 mg/m^2^, Day 1 Every 4–6 weeks plus sorafenib	12.3	33.8	61.5	29.2	21.7	10.8	0.031
		41	Sorafenib	4.9	22.0	41.5	31.7	7.3	8.7	
Kudo et al. [45]	Phase 3 CPS-A, B7	102	Cisplatin 20 mg/m^2^, Day 1, 8 5-FU 330 mg/m^2^ Days 1–5, 8–12 (every 4 weeks) Plus sorafenib	11.7	25.5	56.9	26.5	36.0 (mRECIST)	11.8	0.995
		103	Sorafenib	9.7	21.4	62.1	25.2	18.0 (mRECIST)	11.5	
Zhao et al. [46]	Retrospective CPS-A	46	Oxaliplatin 85 mg/m^2^, Day 1 (every 3 weeks) Plus sorafenib	0	84.8	89.1 (VP3–4)	19.6	34.8	9.4	<0.01
		58	Sorafenib	0	89.7	84.5	27.6	1.7	4.8	
He et al. [47]	Phase 3 PVT CPS-A	125	mFOLFOX 6, Days 1–3 (every 3 weeks) Plus sorafenib	0	80.0	100	30.4	40.8	13.4	<0.01
		122	Sorafenib	0	81.1	100	34.4	2.5	7.1	
**Lenvatinib**										
Mai et al. [48]	Retrospective Single arm	24	mFOLFOX 6, Days 1–3 (every 3 weeks) plus lenvatinib	16.7	10.3	NR	NR	58.3	12 m OS 75%	
**IO-based**										
Gu et al. [49]	Retrospective Single arm	6	mFOLFOX 6, Days 1–3 (every 3 weeks) Apatinib 250 mg QD (since D8) Toripalimab 240 mg D4,	0	NR	100	33.3	100	NR	
He et al. [50]	Retrospective	71	mFOLFOX 6, Days 1–3 Lenvatinib Toripalimab 240 mg per session	0	87.3	77.5	22.5	59.2	NR	<0.001
		86	Lenvatinib	0	90.7	72.1	29.1	9.3	11	
**RT**										
Han et al. [51]	Prospective Single arm PVT	40	5-FU 500 mg/m^2^, Days 1–3 cisplatin 60 mg/m^2^, Day 2 plus RT	0	92.5	100	NR	45	13.1	
Katamura et al. [52]	Retrospective PVT	16	5-FU 500 mg/m^2^, Days 1–5 plus RT	25.0	25.0	100	37.5	75.0	7.5	0.871
		16	5-FU 500 mg/m^2^, Days 1–5	18.8	31.3	100	25.0	25.0	7.9	
Fujino et al. [53]	Retrospective PVT, VP3–4 No EHS	41	cisplatin 20 mg/m^2^, Day 1, 8 5-FU 330 mg/m^2^ Days 1–5, 8–12 INF-α: recombinant 3MU or natural 5MU plus RT	19.5	26.5	100	0	56.1	12.1	0.309
		42	HAIC plus INF-α as above	23.8	23.8	100	0	33.3	7.2	
Kodama et al. [54]	Retrospective PVT and CPS-A, B7	68	Cisplatin 20 mg/m^2^, day 1, 8 5-FU 330 mg/m^2^, Days 1–5, 8–12 (5-FU only in cycle 1–2) plus RT	20.6	29.4	100	19.1	27.8	9.9	0.02
		40	Sorafenib	12.5	42.5	100	40.0	6.7	5.3	

aHCC: advanced hepatocellular carcinoma; CPS: Child–Pugh score; EHS: extrahepatic spread; HAIC: hepatic arterial infusion chemotherapy; HBV: hepatitis B virus; INF-α: interferon-alpha; MVI: macrovascular invasion; mRECIST: modified response evaluation criteria in solid tumors; NR: not reported; ORR: overall response rate; OS: overall survival; PVT: portal vein thrombosis; qW1,3,5: on Monday, Wednesday, Friday every week; QD: every day; QOD: every other day; TACE: transcatheter arterial chemoembolization; VP3: right/left portal vein; VP4: main portal vein; 5-FU: 5-fluorouracil.
